# Wearable and noninvasive monitoring of fluid shifts: A pilot study assessing a novel bioimpedance sensor during lower body negative pressure

**DOI:** 10.14814/phy2.70603

**Published:** 2025-10-05

**Authors:** Lars Øivind Høiseth, Frida Bremnes, Sigve Nyvik Aas, Anne Thorud, Harald Noddeland

**Affiliations:** ^1^ Division of Emergencies and Critical Care Oslo University Hospital Oslo Norway; ^2^ Institute of Clinical Medicine University of Oslo Oslo Norway; ^3^ Mode Sensors AS Trondheim Norway

**Keywords:** diagnostic equipment, edema, fluid shifts, hypovolemia, lower body negative pressure

## Abstract

Fluid extravasation is a prominent feature of several disease processes in acute and critical illness. Bioimpedance at frequencies representing extracellular and total resistance offers an opportunity to monitor fluid shifts. The aim of the present study was to explore if a novel wearable bioimpedance device could monitor fluid shifts during lower body negative pressure (LBNP). Eleven subjects underwent 40 min of LBNP at 30 mmHg. Bioimpedance was measured on the leg, upper back, and thoracic midaxillary line. Changes in resistance were compared using mixed linear regression. Further, hemoglobin and albumin concentrations were measured. Ten subjects completed the protocol. Compared to baseline, both extracellular and total resistance changed to the end of the LBNP exposure at all measurement sites. The mean extracellular resistance changed as follows: leg −6.6% (SD 3.1; *p* < 0.001), upper back 4.1% (SD 3.5; *p* < 0.001), and midaxillary line 6.3% (SD 2.8; *p* < 0.001). The wearable bioimpedance sensor detected the expected extracellular fluid shifts occurring with LBNP. Further clinical studies to explore if fluid extravasation in common pathologic conditions can be measured are warranted.

## 1 INTRODUCTION

Fluid therapy is a crucial aspect of treatment during anesthesia and intensive care. The main objective of maintaining adequate intravascular volume is to ensure optimal cardiac output and global oxygen delivery. However, assessing hydration is challenging due to the distribution of fluids across different body compartments (Armstrong, 2007). Monitoring hydration is important due to its association with disease (El‐Sharkawy et al., 2015), both as a diagnostic tool and to guide therapy. Altered vascular permeability and tissue edema are common, making it potentially valuable to monitor extravascular fluid in a wide range of critically ill patients.

Although frequently used both clinically and experimentally, the terms “dehydration” and “hypovolemia” are not clearly defined. Dehydration is generally understood as a deficit of body water, but both the compartment from which the water is lost as well as the tonicity of the remaining fluid may vary. Hypovolemia refers to a relative or absolute deficiency of intravascular fluid that may occur with or without dehydration (Lacey et al., 2019). The complexity of human fluid and electrolyte homeostasis is reflected by the difficulties in diagnosing hydration and volume status in clinical practice.

Bioimpedance is a non‐invasive technology to assess the fluid content of tissues based on resistance to an applied electrical current. The flow of electrical currents through biological tissues is affected by the current frequency, primarily due to the capacitive properties of cell membranes. As the frequency increases, the current progressively penetrates cell membranes, flowing through both intracellular and extracellular fluids (Earthman et al., 2007). Conversely, as the frequency decreases, current flow becomes increasingly restricted to the extracellular compartment. Measuring impedance over a range of frequencies therefore allows for estimation of the extracellular resistance (*R*_E_) and total resistance (*R*_T_) (Weyer et al., 2012), where *R*_E_ reflects extracellular fluid content and *R*_T_ reflects total fluid content (Armstrong et al., 1997).

Recent advancements in microelectronics have led to the miniaturization of bioimpedance technology, facilitating the development of wearable devices (Groenendaal et al., 2021). These small wearable bioimpedance sensors are limited to measuring specific parts of the body, and the clinical potential of such devices has been insufficiently explored. Before this technology can be used clinically, it is necessary to evaluate its sensitivity to fluctuations in tissue hydration under controlled conditions. Specifically, the extent to which local bioimpedance measurements are affected by changes in various tissue fluid compartments, such as alterations in blood volume, interstitial fluid, and intracellular fluid, should be investigated.

A relevant model for exploring this is lower‐body negative pressure (LBNP). LBNP, originally devised to investigate responses to central hypovolemia (Cooke et al., 2004), involves enclosing the lower body in an airtight chamber where negative pressure is applied, leading to blood displacement towards the lower extremities and pelvis. Although differing somewhat between studies, it is estimated that an LBNP of 30 mmHg corresponds to a blood loss of approximately 450 mL (Goswami et al., 2019; Hinojosa‐Laborde et al., 2013). The movement of blood is rapid, and compensatory responses are triggered by reduced venous return to the heart and reduced vascular filling in the upper part of the body. Baroreceptors are stimulated and cause general vasoconstriction (Goswami et al., 2019). Heart rate is increased and diminishes the reduction in arterial blood pressure and cardiac output (Cooke et al., 2004). The responses are quick, and within a few minutes of moderate LBNP, a new steady state is obtained securing cerebral perfusion. When hypovolemia is maintained for a longer period, another and slower acting compensatory mechanism is activated: mobilization of extracellular fluid from the interstitium to plasma (Lundvall et al., 1993). Within the LBNP chamber, an initial increase in leg volume is caused by an increase in the intravascular venous compartment (Coles et al., 1957). Thereafter, prolonged LBNP will displace fluid to the interstitial tissues (Aratow et al., 1993), resulting in lower body edema (Lindenberger & Länne, 2007). A previous study employing a stationary bioimpedance device during LBNP reported increased upper‐body impedance and reduced lower‐body impedance, supporting the suitability of this technology for detecting fluid shifts (Anakmeteeprugsa et al., 2024).

The objective of the current investigation was to investigate the ability of a novel wearable bioimpedance device (Noddeland et al., 2024) to track both immediate and slow‐occurring displacement of blood and fluid induced by LBNP in healthy volunteers. As no changes in electrolytes, glucose, and other osmotic substances acting across the cellular membrane are expected, the total cellular volume, including total erythrocyte volume, is assumed to remain unchanged. It is therefore anticipated that movement of fluid will primarily affect the extracellular compartment (plasma volume and interstitial fluid volume). Hence, it was hypothesized that LBNP would result in (1) decreased extracellular resistance (*R*_E_) in the lower body, and (2) increased *R*_E_ in the upper body.

## 2 MATERIALS AND METHODS

The study was performed at Oslo University Hospital in September 2023. The investigation was registered in clinicaltrials.gov prior to subject recruitment (NCT06003205) and was approved by the Regional Committees for Medical and Health Research Ethics in Norway (REK KULMU, ref. 606538) and the Norwegian Medical Products Agency (NOMA).

### 2.1 Study population

Subjects were included after written informed consent. Healthy volunteers between 18 and 50 years were eligible for inclusion after assessment of the subject's medical history and a focused cardiac ultrasound. Exclusion criteria were (1) known allergies or skin sensitivities to electrode hydrogel and/or acrylic adhesives, (2) breached skin at the device application area, (3) implantable pulse generators such as pacemakers and defibrillators, and/or use of other electrical medical equipment for which an interaction effect with the investigational device could not be ruled out, (4) pregnancy, (5) breastfeeding, (6) history of syncope, (7) any medical condition limiting physical capacity, (8) any known cardiac disease that, at the investigator's discretion, warranted exclusion from the study.

### 2.2 Investigational device

The bioimpedance device used in the investigation (Re:Balans®, Mode Sensors AS, Trondheim, Norway) is a lightweight (<15 g), flexible, low‐profile sensor patch. The investigational device has been described in detail previously (Noddeland et al., 2024). The device performs bioimpedance measurements periodically (every 30 s by default) but was configured to sample at 10‐s intervals for this study. During each measurement cycle, the impedance is measured at 32 distinct frequencies from 1 kHz to 1 MHz. The absolute impedance measurements are frequency dependent and are used to estimate the extracellular (*R*_E_) and total resistance (*R*_T_) by a model‐based approach analogous to the well‐known Cole–Cole model (Cole & Cole, 1941). The device and model have been used in two previously published clinical investigations (Bremnes et al., 2025; Noddeland et al., 2024). The measurements are subject to a series of objective quality assurance (QA) checks (e.g., signal‐to‐noise ratio), and the measurement accuracy is reported by the manufacturer to be within ±2% compared to reference loads.

With four electrodes (tetrapolar set‐up) the skin‐electrode contact impedance has limited impact on the measured impedance. Based on the distance between the electrodes of the investigational device, most of the measured impedance reflects tissue within a depth of approximately 5 cm (Jafarpoor et al., 2013; Kassanos, 2021). Hence, the device is sensitive to fluid volume changes not only in the skin (dermis) but also deeper tissue layers (e.g., subcutaneous adipose tissue and muscle).

**FIGURE 1:**
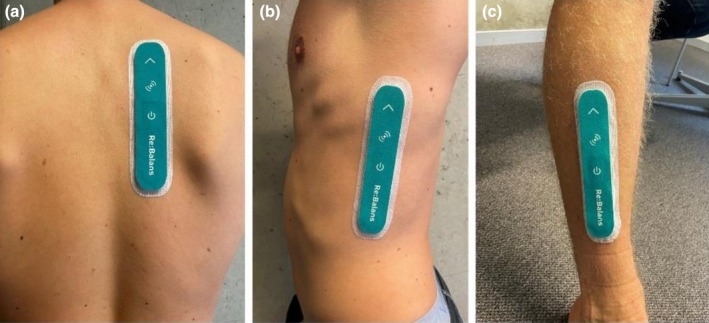
The investigational device at the upper back (a), midaxillary line (b), and lower leg (c). The subject consented to the publication of this anonymised image.

Each subject was monitored by three sensor devices placed on three separate anatomical locations: upper back, midaxillary line, and lower leg. At the upper back, the investigational device was placed 2–4 cm paravertebral left or right, at the height between the shoulder blades. This placement has been used in two previous clinical investigations (Bremnes et al., 2025; Noddeland et al., 2024). The device placed along the midaxillary line was placed between the fourth and 12th rib (anterior to m. latissimus dorsi and posterior to m. pectoralis major). This placement was included to obtain data on an upper body placement that is not exposed to physical compression during the LBNP procedure (supine position). The third device was placed on the lower leg, over m. tibialis anterior (anterolateral placement). This placement was chosen because it corresponds to a site where LBNP‐induced fluid accumulation was anticipated and where the sensor is not exposed to physical compression in the supine position. Additionally, it was selected to evaluate the ability of the device to detect peripheral fluid accumulation, which is relevant for several patient groups. The investigational device and the three placements are shown in Figure 1. The investigational device was removed the day after the LBNP protocol.

### 2.3 Signal processing

After quality assurance, the data was interpolated and filtered using a low‐pass symmetric FIR filter to attenuate high‐frequency changes (e.g., temporary changes caused by muscle activity). The timing of the signal was adjusted for delays introduced by the filtering process before further analysis.

### 2.4 Study procedures

**FIGURE 2:**
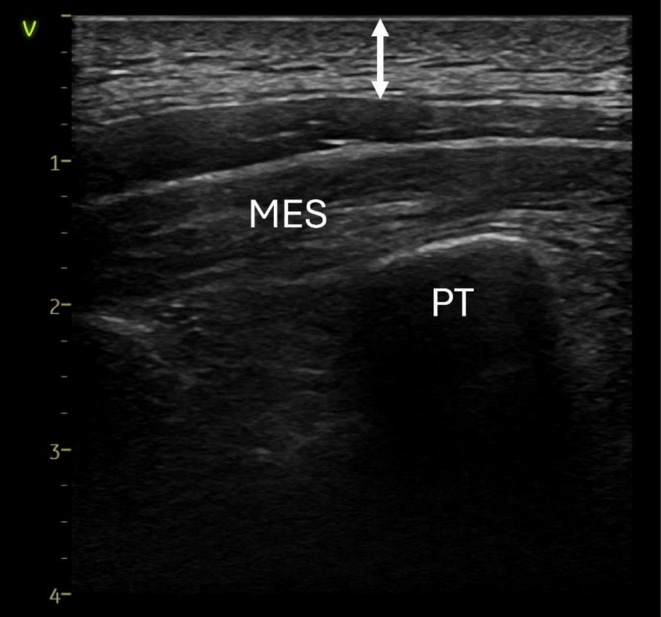
Example of ultrasound measurements of skin and subcutaneous thickness from the upper back. The measured distance from the skin surface to the most superficial muscular layer marked by the white arrow. The scale on the vertical axis is centimeters. The subject consented to publication of this anonymised image. PT, processus transversus; MES, musculus erector spinae.

On the day of the LBNP intervention, subjects underwent initial assessments including height and weight. Skin thickness at the upper back and midaxillary line placements was assessed by ultrasound (Vivid E95; GE Healthcare, Chicago, USA). A generous amount of ultrasound gel was applied to allow for minimal pressure on the skin when acquiring the ultrasound images. The measurement of skin thickness by ultrasound includes epidermis, dermis, and hypodermis (subcutaneous adipose tissue). All image acquisitions and measurements were conducted by a single operator (LØH), an anesthesiologist with routine experience in clinical ultrasound. The vertical distance, measured perpendicular to the skin surface, was taken from the skin to the most superficial muscular layer using the software of the ultrasound machine (EchoPAC 202, GE Vingmed, Horten, Norway). An example of the ultrasound measurements of skin thickness from the upper back is presented in Figure 2.

Skin thickness at the upper back was also assessed by a skinfold caliper (Slim Guide, Creative Health Products, USA). Photographs were taken to document skin condition prior to device exposure. Sensors were then applied and activated, and a new photograph was obtained to document the exact placement of the device.

The LBNP apparatus used in this investigation was a custom‐built device located at the Section of Vascular Investigations, Oslo University Hospital‐Aker, Oslo, Norway. The device has been described in detail previously (Hisdal et al., 2003). In short, subjects are placed supine in the LBNP chamber sealed at the iliac crest. A “saddle” is placed in the chamber to prevent caudal displacement during LBNP and contraction of leg muscles (Hisdal et al., 2003). LBNP was relieved upon occurrence of any of the following: symptoms of pre‐syncope, reduction in mean arterial pressure or heart rate to <75% of baseline values for >3 s or subject request. Pressure inside the chamber was continuously monitored. The LBNP setup has been used in several investigations without any serious adverse events.

**FIGURE 3:**
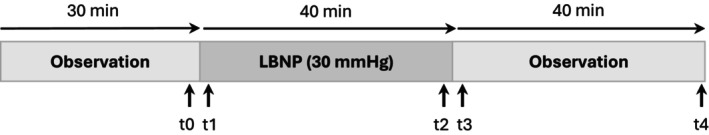
Sequence of events in the LBNP intervention. t0: 2 min before LBNP was turned on, t1: 2 min after LBNP was turned on, t2: 2 min before LBNP was turned off, t3: 2 min after LBNP was turned off, t4: 38 min after LBNP was turned off.

The subjects stayed in a supine position throughout the whole experiment, and the sequence of events is shown in Figure 3. Following an observation period of 30 min, subjects were exposed to 30 mmHg LBNP for 40 min. LBNP exposure was followed by another 40 min observation period. Timepoints used for statistical analysis were defined as follows: t0 (baseline) corresponds to 2 min prior to the onset of LBNP; t1 corresponds to 2 min after the onset of LBNP; t2 and t3 correspond to 2 min before and after the end of LBNP exposure, respectively; and t4 corresponds to 2 min before the end of the observation period. At all timepoints, 1‐min averages were used for analyses.

In addition to the continuous bioimpedance measurements by the investigational device, several other physiological parameters were monitored throughout the intervention. Arterial blood pressure was measured by the volume‐clamp method (Nexfin; Edwards Lifesciences Corp., CA, USA), which also gave stroke volume by pulse contour analysis. This was sampled together with ECG/heart rate (BioAmp/Powerlab; AD Instruments, Dunedin, New Zealand) and arterial oxygen saturation (Masimo Radical 7; Maximo Corp., CA, USA) at 1000 Hz in LabChart v8.1.28. Measurements were down‐sampled to average values for each heartbeat for further analysis.

A blood sample was obtained at t0, t3, and t4 and analyzed for hemoglobin, erythrocyte volume fraction (EVF), and albumin. Total blood volume was estimated using the Nadler Equation (Nadler et al., 1962), and hemoglobin and EVF values were used to calculate changes in blood volume, plasma volume, and red cell volume (Dill & Costill, 1974).

### 2.5 Statistical analyses

Baseline data, absolute and relative changes are expressed as mean ± standard deviation or median (range) unless otherwise stated. For comparative analyses, a mixed effects linear model was used. Subjects were treated as random effects to account for variability across observations, while the timepoint was treated as a factor. The modeling and contrast testing were performed in R Statistical Software (v 4.4.0 R Core Team (2024)) using the packages *nlme* and *multcomp*, respectively (Hothorn et al., 2008; Pinheiro & Bates, 2023).

Reference data and bioimpedance data at intervention timepoints t1, t2, t3 and t4 were compared to baseline (t0). Additionally, the period during the LBNP exposure (t1–t2), the immediate effect of turning the LBNP off (t2–t3), and the observation period (t3–t4) were included in the analysis. The “lme” function from the *nlme* package was employed for mixed‐effects linear modeling, and overall significance was assessed for each model using the “anova” function from the *nlme* package. Contrast testing was performed if the overall test was significant by using the “glht” function from the *multcomp* package with a “single‐step” adjustment of *p*‐values. Data processing and additional analyses were performed using Python (v 3.12) with packages *NumPy, SciPy, pandas*, and *statsmodels* (Harris et al., 2020; Seabold & Perktold, 2010; The pandas development team, 2020; Virtanen & Gommers, 2020). For correlation analyses examining the relationship between absolute *R*_E_ and skin thickness, *R*_E_ was averaged over a 24‐h measurement period spanning from t0 to 24 h post t0. This time window was chosen to mitigate the influence of short‐term fluctuations and transient experimental effects, which are expected to average out during the 24‐h time window. The average value was calculated using only data points that passed the quality assurance criteria. A *p*‐value *p* < 0.05 was considered statistically significant for all tests.

### 2.6 Sample size calculation

The minimum sample size was calculated based on the primary endpoint, which was the relative change in *R*_E_ of the calf from start to end of LBNP. A change of 3% was considered clinically significant. The standard deviation was assumed to be of a similar magnitude as the average effect due to the highly controlled setting of the LBNP experiment, giving an effect size of 1 (mean difference/standard deviation). The sample size was then calculated using the formula

$$n={\left(\frac{Z_{1-\alpha /2}+Z_{1-\beta }}{\delta }\right)}^{2}$$

where $Z_{1-\alpha /2}$ is the critical value corresponding to the chosen significance level, $Z_{1-\beta }$ is the critical value corresponding to the desired power, and δ represents the effect size. With a desired significance level of 0.05 and power of 80%, we found that a minimum of 8 participants was required.

## 3 RESULTS

Eleven subjects were recruited for the investigation, and subject characteristics are presented in Table 1.

**TABLE 1:** Subject characteristics. Data are *n* or median (range).

Men/Women, *n*	3/8
Age, years	33 (23–48)
Weight, kg	71 (50–107)
Height, cm	172 (152–182)
Body mass index, kg × m^−2^	24 (21–35)

Ten subjects completed the LBNP intervention according to the protocol. One subject experienced discomfort during the LBNP procedure, and the LBNP was stopped after 11 min. Accordingly, this subject was excluded from all analyses directly associated with the LBNP experiment. Mean values for all measured reference parameters at timepoints t0–t4 are presented in Table 2.

**TABLE 2:** Mean values before (t0), during (t1 and t2), and after (t3 and t4) LBNP.

Timepoint	Baseline	LBNP		Observation	
	t0	t1	t2	t3	t4
MAP, mmHg	94 ± 16	96 ± 14	95 ± 14	96 ± 15	96 ± 18
Stroke volume, mL	100 ± 11	93 ± 13*	87 ± 9*	98 ± 11	97 ± 12
Heart rate, beats × min^−1^	60 ± 8	60 ± 6	66 ± 6*	62 ± 8	61 ± 8
Cardiac output, L × min^−1^	5.9 ± 1.0	5.5 ± 0.5	5.7 ± 0.8	6.1 ± 1.2	6.0 ± 1.2
Arterial O_2_ saturation, %	98 ± 1.8	99 ± 1.7	97 ± 1.8*	97 ± 2.1*	98 ± 1.7
Albumin, g × L^−1^	44.1 ± 2.1	‐	‐	46.3 ± 2.0*	44.4 ± 2.2
Hemoglobin, g × dL^−1^	13.3 ± 1.3	‐	‐	13.9 ± 1.2*	13.5 ± 1.1
EVF	0.41 ± 0.03	‐	‐	0.43 ± 0.03*	0.42 ± 0.03*

* Significant change from t0 (*p* < 0.05).

The initial effect of exposure to LBNP at 30 mmHg (t0‐t1) was a decrease in stroke volume. During the 40 min of LBNP exposure (t1‐t2) the stroke volume decreased further while the heart rate increased. Compared to t0, the cardiac output tended to be lower at the end of the exposure period (t2), but this difference was not significant.

Shortly after turning the LBNP off (t2–t3), there was an increase in stroke volume and a decrease in heart rate, as both were returning to baseline values. The stroke volume and heart rate then remained stable for the rest of the observation period.

Arterial oxygen saturation was not acutely affected by changes in LBNP but decreased slightly during the LBNP exposure (t1‐t2) and increased back to initial levels during the observation period (t3–t4).

Blood samples taken shortly after the LBNP was turned off (t2) revealed an increase in albumin, hemoglobin, and EVF compared to t0. Albumin, hemoglobin, and EVF decreased during the observation period (t3–t4). EVF remained slightly elevated at t4 compared to t0. A similar trend was noted for albumin and hemoglobin, but the difference between t0 and t4 was not significant.

### 3.1 Bioimpedance measurements

**FIGURE 4:**
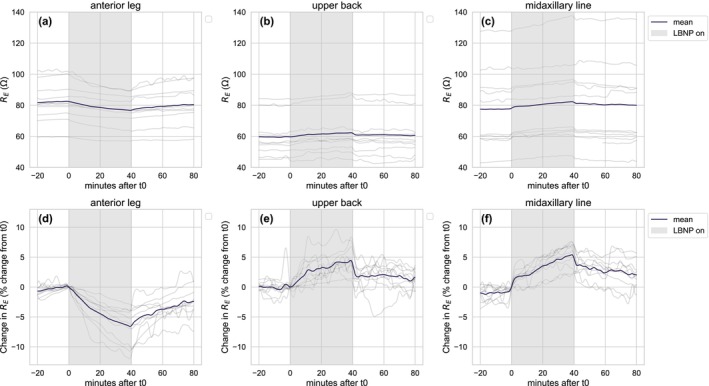
Absolute (upper panels) and relative changes (lower panels) in *R*_E_ during the LBNP intervention at the lower anterior leg (a and d), upper back (b and e), and midaxillary placement (c and f). Individual values are shown (gray lines) together with the average response (black lines) (*N* = 10 participants included).

All investigational devices met the pre‐defined quality threshold for inclusion in analyses. Absolute and relative changes in extracellular resistance (*R*_E_) at the three sensor device placements are presented in Figure 4. A marked response to LBNP was observed at all three anatomical sites.

Absolute and relative changes in both extracellular resistance (*R*_E_) and total resistance (*R*_T_) are presented in Table 3.

**TABLE 3:** Absolute and relative changes in extracellular resistance (*R*_E_) and total resistance (*R*_T_) at the upper back, lateral thorax, and anterior leg. Mean values are presented for timepoints before (t0), during (t1 and t2), and after (t3 and t4) LBNP exposure. Values are mean ± SD.

	Baseline	LBNP		Observation	
	t0	t1	t2	t3	t4
Anterior leg					
*R*_E_, Ω	82.5 ± 12.1	82.1 ± 12.2	76.8 ± 10.0*	77.5 ± 10.6*	80.6 ± 12.2
*R*_T_, Ω	41.0 ± 13.7	40.9 ± 13.8	39.0 ± 12.5*	39.4 ± 12.6*	40.4 ± 13.2
*R*_E_, %	0 ± 0	−0.6 ± 0.7	−6.6 ± 3.1*	−5.9 ± 3.2*	−2.4 ± 2.5*
*R*_T_, %	0 ± 0	−0.3 ± 1.4	−4.1 ± 3.3*	−3.1 ± 3.6*	−0.9 ± 3.2
Upper back					
*R*_E_, Ω	59.8 ± 12.2	59.8 ± 12.7	62.4 ± 13.5*	60.9 ± 13.5	60.6 ± 13.2
*R*_T_, Ω	36.0 ± 14.5	36.5 ± 14.4	38.2 ± 15.3*	37.3 ± 15.1*	37.1 ± 15.1*
*R*_E_, %	0 ± 0	−0.1 ± 2.5	4.1 ± 3.5*	1.5 ± 3.0	1.0 ± 3.0
*R*_T_, %	0 ± 0	1.6 ± 3.4	6.4 ± 4.2*	3.6 ± 4.0*	2.6 ± 5.2
Midaxillary line					
*R*_E_, Ω	77.6 ± 24.6	79.1 ± 24.2	82.3 ± 25.7*	81.0 ± 25.7*	80.1 ± 26.3*
*R*_T_, Ω	55.2 ± 26.6	56.3 ± 26.5	58.6 ± 27.6*	57.7 ± 27.5*	57.0 ± 28.2*
*R*_E_, %	0 ± 0	2.2 ± 2.0	6.3 ± 2.8*	4.4 ± 1.7*	2.8 ± 2.4*
*R*_T_, %	0 ± 0	2.3 ± 3.3	7.0 ± 4.2*	4.9 ± 1.8*	2.4 ± 4.0

* Significant change from t0 (*p* < 0.05).

The total effect of the 40‐min exposure period was both absolute and relative changes in *R*_E_ and *R*_T_ at all sensor placements. *R*_E_ and *R*_T_ increased at both upper body placements (midaxillary line and upper back) and decreased at the anterior leg placement during the exposure period (t1–t2). There were no significant immediate effects of turning the LBNP on (t0–t1), although an insignificant increase in *R*_E_ and *R*_T_ at the midaxillary line placement was noted together with a slight increase in *R*_T_ at the upper back placement.

When the LBNP was turned off (t2–t3), there was an immediate absolute and relative decrease in *R*_E_ measured at the upper back. A similar but insignificant decrease was noted for the midaxillary line placement. At the end of the observation period (t4), the measurements were still slightly higher at the upper back and midaxillary line placements and lower at the anterior leg placement when compared to t0. However, this difference was only significant for some of the timepoints and variables.

**FIGURE 5:**
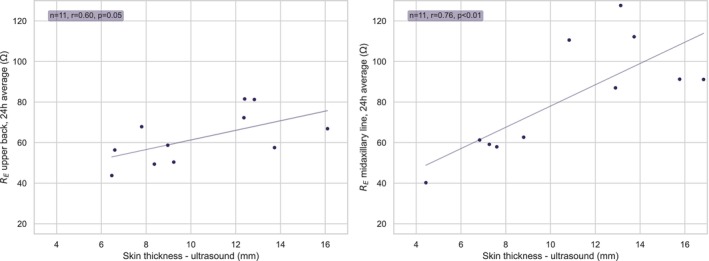
The figure displays the correlation between skin thickness measured using ultrasound at the upper back (left) and midaxillary line (right) and *R*_E_ measured at the same location, averaged over 24 h. The correlation includes all subjects (*N* = 11).

A large interpersonal variation in absolute values of *R*_E_ was observed (Figure 4, upper panel). Figure 5 displays correlations between skin thickness measured by ultrasound at the upper body placements (upper back and midaxillary line), and R_E_ averaged over 24 h at the same placements. At the midaxillary line placement, a strong positive correlation was observed between skin thickness measured by ultrasound and *R*_E_ (*r* = 0.76, *p* < 0.01). The upper back also displayed a positive trend, but the correlation was not statistically significant (*r* = 0.60, *p* = 0.05). However, skin thickness assessed by fat caliper at the upper back was found to have a strong positive correlation with *R*_E_ averaged over 24 h (*r* = 0.75, *p* < 0.05).

An example of the ultrasound measurements of skin thickness from the upper back is presented in Figure 2. *R*_T_ averaged over 24 h also had a strong correlation with skin thickness assessed by ultrasound at both sites (*r* = 0.81 and *p* < 0.01 for the midaxillary line, *r* = 0.72 and *p* < 0.05 for the upper back). Skin thickness assessed by fat caliper also displayed a strong correlation with *R*_T_ (0.86, *p* < 0.01). Hence, absolute *R*_E_ and *R*_T_ tended to be higher in subjects with greater skin thickness.

### 3.2 Changes in circulating blood

Estimated changes in blood volume, red cell volume, and plasma volume on timepoints t0, t3, and t4 are presented in Table 4. Exposure to LBNP was followed by a decrease in plasma and blood volume at timepoint t3. The reduction in blood volume was primarily attributed to the decrease in plasma volume. Although plasma volume increased during the observation period from t3 to t4, it remained lower at t4 compared to t0. Red cell volume showed no significant changes throughout the experiment.

**TABLE 4:** Absolute and relative changes in blood composition, from t0 (before LNBP) to t3 (immediately after LBNP cessation) and t4 (40 min after LNBP cessation). Estimates are based on hemoglobin and EVF values (presented in Table 2) and established equations (Dill & Costill, 1974; Nadler et al., 1962).

Timepoint	Baseline	LBNP		Observation	
	t0	t1	t2	t3	t4
Blood volume, mL	4517 ± 830	‐	‐	4346 ± 868*	4461 ± 855
Blood volume, %	0 ± 0	‐	‐	−4.0 ± 1.8*	−1.3 ± 1.9
Plasma volume, mL	2651 ± 371	‐	‐	2474 ± 399*	2582 ± 374*
Plasma volume, %	0 ± 0	‐	‐	−6.9 ± 2.5*	−2.6 ± 2.8*
Red cell volume, mL	1866 ± 487	‐	‐	1871 ± 495	1879 ± 496
Red cell volume, %	0.0 ± 0.0	‐	‐	0.3 ± 1.8	0.7 ± 1.5

* Significant change from t0 (*p* < 0.05).

### 3.3 Safety and user tolerance

All 11 subjects were included in the safety analysis. The investigational device exposure time was 28 ± 2 h. No device‐related adverse events were reported. These results agree with the risk assessment conducted prior to study initiation, confirming that the device is well tolerated.

None of the subjects experienced serious adverse events during LBNP exposure. Although one subject experienced discomfort compatible with pre‐syncope, this is to be expected in the LBNP model and is in fact often used as an end point (Goswami et al., 2019). The research group performing this study has extensive experience with the LBNP‐model in healthy volunteers, studying at least 120 subjects in the last 10 years without experiencing serious adverse events.

## 4 DISCUSSION

The main finding in this study was that a small and wearable bioimpedance sensor can track fluid displacement induced by LBNP in healthy volunteers. This finding corresponds well with findings from a previous study conducted using stationary bioimpedance equipment (Anakmeteeprugsa et al., 2024). A consistent decrease in extracellular resistance (*R*_E_), accompanied by a slightly smaller decrease in total resistance (*R*_T_), was observed at the lower leg of all included subjects following 40 min of negative pressure. This finding aligns well with the expected LBNP‐induced increase in lower leg extracellular fluid volume. The decrease in *R*_E_ and *R*_T_ at the lower leg was mirrored by consistent increases at both the upper back and midaxillary line placements, indicative of a reduction in extracellular fluid volume at these anatomical sites.

**FIGURE 6:**
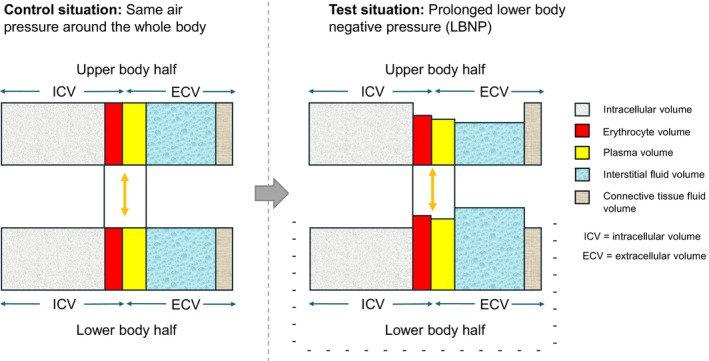
Illustration of fluid compartments in the upper and lower body half in a control situation with the same air pressure around the whole body (left panel) and following prolonged negative air pressure around the lower half of the body (right panel).

The hemodynamic findings were as expected according to earlier studies (Goswami et al., 2019), with the mild LBNP being associated with a reduction in stroke volume and a slight increase in heart rate, whereas MAP was maintained. Cardiac output was not reduced significantly, as the reduction in stroke volume was nearly compensated by a slight increase in heart rate at the end of LBNP. The redistribution of blood occurs rapidly following LBNP onset, and primarily within the venous compartment (Goswami et al., 2019). In the present investigation, where a pressure of −30 mmHg was applied, we can assume that approximately 450 mL of blood was redistributed rapidly from the upper body to the lower extremities (Goswami et al., 2019; Hinojosa‐Laborde et al., 2013). Prolonged exposure has been shown to elicit greater changes in transcapillary filtration, both in the upper and lower body. In one study (Aratow et al., 1993), 4 h of LBNP at 30 mmHg was found to decrease leg interstitial fluid pressure, increase movement of fluid to the leg, and increase leg circumference, without significant changes in whole body net transcapillary fluid transport. Another study (Lundvall et al., 1993) using LBNP at 70–75 mmHg observed filtration of fluid to the legs leading to edema with compensatory absorption of extravascular fluid from the upper body. Although these studies differ from ours in length and/ or LBNP‐intensity, the LBNP‐model seems to initially shift blood from the upper to the lower body, whereas prolonged exposure also leads to a shift in extravascular fluid. A proposed illustration of the expected fluid shifts occurring during prolonged LBNP is presented in Figure 6.

In the upper body, the hydrostatic pressure in capillaries and small veins is reduced, while the plasma colloid osmotic pressure in circulating blood is expected to increase due to increased transcapillary filtration in the lower body. The result is a transcapillary shift of fluid from the interstitial space to the plasma (Lundvall et al., 1993). The expected response is well reflected in our data, as we observed increased *R*_E_ both at the upper back and on the midaxillary placement following 40 min of LBNP, in addition to an increase in albumin concentration.

In the lower body, the interstitial fluid hydrostatic pressure is reduced by the applied negative pressure, while the hydrostatic capillary pressure gradient is increased, leading to the displacement of blood and increased vascular filling. The result is an increased net filtration pressure despite a moderately increased plasma colloid osmotic pressure. The expected effect is a transcapillary shift of extracellular fluid from plasma to the interstitium, eventually leading to skin edema (Aratow et al., 1993; Lundvall et al., 1993). This is also well reflected in our data, as we observed significantly reduced *R*_E_ at the lower leg following prolonged LBNP.

One objective of the current investigation was to compare immediate versus delayed responses, to obtain preliminary information on the relative impact of blood displacement and transcapillary filtration. This was investigated based on the assumption that the immediate effect of LBNP is a displacement of blood, whereas prolonged LBNP to a greater extent induces transcapillary transport between the vascular compartment and the interstitial space. An interesting observation in this regard was that the extent of immediate versus delayed impedance responses was dependent on sensor placement. At the lower leg, there was no immediate change in *R*_E_ and *R*_T_ when LBNP was turned on and off (Figure 4d). Changes in impedance only occurred *during* the negative pressure procedure, perhaps reflecting that this sensor placement is mostly sensitive towards changes in the interstitial fluid volume of the tissue. It is nevertheless possible that gradually increased pooling of blood in the vasculature also contributed to the gradual decrease in impedance.

At the midaxillary placement, both an immediate (albeit statistically non‐significant) and delayed change in *R*_E_ and *R*_T_ was observed (Figure 4f, Table 3). The immediate change may reflect a rapid decrease in thoracic blood volume (Goswami et al., 2019). The delayed response (t1–t2) may to a greater extent reflect fluid transport from the interstitial to the vascular space. Indeed, absorption of fluid in the upper body during LBNP has been observed previously (Aratow et al., 1993; Länne & Lundvall, 1989), and is likely driven in part by an increase in the plasma oncotic pressure (Aratow et al., 1993). This is supported by the observed increase in albumin in the present study. LBNP has also been shown to elicit vasoconstriction in the upper body due to baroreceptor unloading, which may reduce capillary pressure, also favoring upper body fluid reabsorption (Johnson et al., n.d.).

The results for the upper back placement are somewhat more difficult to interpret. No immediate effect was observed when pressure was turned on, but a non‐significant immediate change was observed when the pressure was released (Figure 4e). A gradual increase in both *R*_E_ and *R*_T_ was observed *during* LBNP. It is noted that the measurements at the upper back displayed larger fluctuations throughout the intervention. Since the subjects were lying directly on the upper back sensor, physical compression of the tissue at this site is likely to have influenced the recordings. Such compression could alter local tissue resistance and contribute to the observed variability. In addition, small subject movements may have caused shifts in the applied pressure as well as minor skin displacement relative to the underlying muscle tissue. Taken together, these factors indicate that the results for the upper back location should be interpreted with caution.

When the LBNP was turned off, the change in *R*_E_ and *R*_T_ was reversed at all sensor placements. However, *R*_E_ was still significantly different from baseline at the lower leg and midaxillary placement 40 min after LBNP had been relieved. These results indicate that interstitial fluid volume was still increased at the lower leg and decreased at the midaxillary placement after the observation period. This is also supported by the fact that neither albumin, hemoglobin, EVF, nor plasma volume had returned to pre‐LBNP levels at this timepoint.

As shown in the top panel in Figure 4, a large interpersonal variation in the absolute impedance measurements was observed. Adipose tissue is a poor electrical conductor, and the thickness of the subcutaneous adipose tissue (SAT) layer will have a significant impact on local bioimpedance measurements (Jafarpoor et al., 2013). The correlation observed between skin thickness and absolute *R*_E_ and *R*_T_ values in the present study is therefore most likely related to variation in SAT thickness. The variation was greatest at the midaxillary placement, in line with the notion that SAT thickness varies significantly at this anatomical site. Despite the substantial variation in absolute values, it is noteworthy that the response to LBNP was very consistent across subjects. In sum, these results indicate that the device is capable of tracking relative changes in fluid status at several anatomical sites.

Fluid therapy is one of the most performed therapeutic actions in acute and critically ill patients. Increased vascular permeability may increase the displacement of fluids from the intravascular to the extravascular space in conditions such as sepsis, burns, anaphylaxis, and post‐cardiopulmonary bypass (Dargent et al., 2023; Krishnaswamy, 2021; Lund et al., 1988). This has been termed capillary leak syndrome (Saravi et al., 2023) and contributes not only to tissue oedema but may also lead to intravascular hypovolemia and global hypoperfusion if intravascular fluids are not replaced. Whereas much focus has been rightly placed on methods to titrate fluids to ensure adequate amounts of intravascular fluid (Benes et al., 2015), for example, by assessing fluid responsiveness, the assessment of tissue oedema has received much less attention. In this regard, a compact and wearable bioimpedance sensor may create new possibilities within patient care by enabling continuous monitoring of tissue oedema.

In conclusion, the fluid shifts induced by LBNP in healthy volunteers were associated with a decrease in both extracellular and total resistance at the leg placement, whereas both resistances increased on the upper back and midaxillary line. These results indicate that the investigational device is able to track the changes in extracellular fluid volume induced by LBNP. Further studies on the ability of the device to track fluid shifts in clinical scenarios where such shifts are of significance, for example, sepsis, are warranted. The results presented in this article align with previous research demonstrating the device's ability to detect changes in fluid volume in a controlled setting, both in healthy volunteers (Noddeland et al., 2024) and in patients with kidney failure (Bremnes et al., 2025). Further research should explore the ability of the sensor to measure fluid changes over longer periods and in less controlled environments. Such data are crucial for assessing the device's performance beyond controlled experimental settings and understanding its potential clinical utility across diverse patient populations and conditions.

## AUTHOR CONTRIBUTIONS

Lars Øivind Høiseth, Frida Bremnes, Sigve Nyvik Aas, and Harald Noddeland contributed to the conception of the study. Data collection was performed by Lars Øivind Høiseth and Anne Thorud. Lars Øivind Høiseth, Frida Bremnes, Sigve Nyvik Aas, and Harald Noddeland contributed to data analyses and interpretation of results. Lars Øivind Høiseth, Frida Bremnes, Sigve Nyvik Aas, and Harald Noddeland drafted the manuscript. All authors revised the manuscript and approved its final version.

## FUNDING INFORMATION

The clinical investigation was funded by the Research Council of Norway (grant number 313922).

## CONFLICT OF INTEREST STATEMENT

Lars Øivind Høiseth, Harald Noddeland, and Anne Thorud declare no competing interests. Frida Bremnes and Sigve Nyvik Aas are employed by Mode Sensors AS, the company developing the sensor tested in the clinical investigation. Frida Bremnes and Sigve Nyvik Aas have stock options in the company. Frida Bremnes and Sigve Nyvik Aas were not present during data collection but contributed to study design, data analysis, interpretation, and manuscript preparation.

## ETHICS STATEMENT

The study was approved by the Regional Committees for Medical and Health Research Ethics in Norway (REK KULMU, ref. 606538) and the Norwegian Medicines Agency (NoMA). The study was conducted in accordance with the Declaration of Helsinki and ISO14155:2020–Good clinical practice. Written informed consent was obtained from each participant prior to study enrolment.

## CONSENT

Not applicable.

### TRIAL REGISTRATION

The study was registered in clinicaltrials.gov prior to recruitment of subjects (NCT06003205).

## Data Availability

The datasets generated in the study are available from the corresponding author upon reasonable request.
